# Heart failure in the elderly: epidemiology, mechanisms, and management

**DOI:** 10.1093/eurheartj/ehag110

**Published:** 2026-02-28

**Authors:** Rudolf A de Boer, Mahmoud Abdellatif, Johann Bauersachs, Veronique L Roger

**Affiliations:** Department of Cardiology, Thorax Center, Cardiovascular Institute, Erasmus MC, Dr. Molewaterplein 40, Rotterdam 3015GD, The Netherlands; Department of Cardiology, Medical University of Graz, Graz, Austria; Department of Cardiology and Angiology, Hannover Medical School, Hannover, Germany; National Heart Blood and Lung Institute, National Institutes of Health, Bethesda, MD, USA

**Keywords:** Heart failure, Ageing, Pharmacotherapy, Device therapy, Autophagy, Mitochondria, Palliative care

## Abstract

There is no consensus on an age cut-off for being considered elderly, but the majority of patients with heart failure (HF) have an advanced age. The lifetime risk for developing HF is ∼25%, with a sharp increase in incidence after the age of 70. The lifetime risk for men and women is almost equal, but women exhibit a higher propensity towards developing HF with preserved ejection fraction, whereas men are more prone to HF with reduced ejection fraction. During the biological ageing process, several systemic and local pathophysiological alterations impact the myocardium, including impaired autophagy and proteostasis, mitochondrial dysfunction, and oxidative stress, as well as cellular senescence, clonal haematopoiesis of indeterminate potential, and chronic low-grade inflammation or inflammaging. Collectively, these changes compromise cardiac energy homeostasis and promote cell loss and dysfunction, increasing the risk of HF. Despite their relevance, these ageing-related mechanisms are hitherto not addressed by guideline-recommended medical therapy. Guideline-recommended medical therapy remains the cornerstone of HF treatment across age groups, including in elderly patients who tolerate it. However, a high burden of comorbidities and several features specific to advanced age, such as low blood pressure and frailty, often preclude full-dose guideline-recommended medical therapy. Similarly, the risk–benefit ratio of device therapies needs careful consideration in light of competing non-cardiac risks due to comorbidities that are prevalent in this population. Finally, HF is a mortal condition, and advanced care planning and end-of-life decisions should be discussed in a timely manner in elderly patients.

## Graphical Abstract

**Graphical Abstract:**
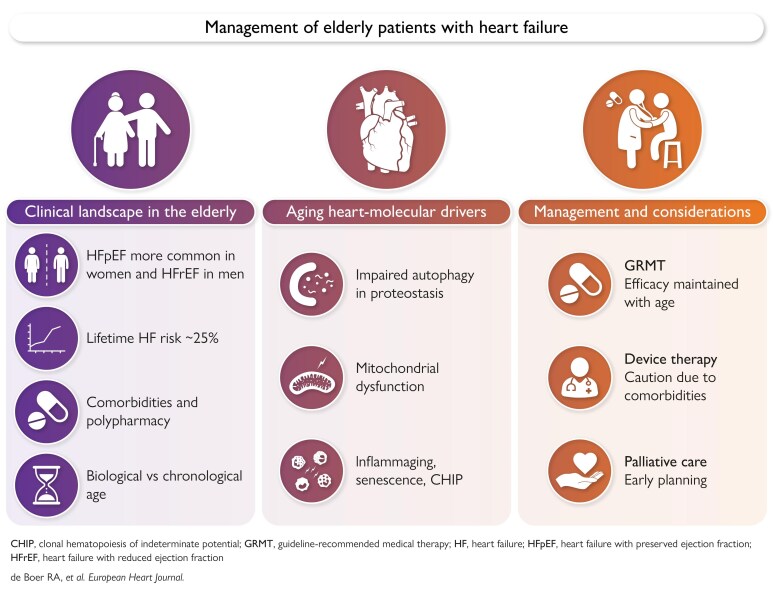
Heart failure is a common condition especially affecting the elderly. Lifetime risk is substantial, and specific subforms are dominant in men and women. The ageing heart is characterized by specific molecular abnormalities, including impaired autophagy and proteostasis, mitochondrial dysfunction, inflammation, senescence, and clonal haematopoiesis of indeterminate potential (CHIP) as main drivers. Despite challenges with dosing and more frequent side effects, guideline-recommended medical therapy (GRMT) is advised in the elderly as in all other patients.

## Introduction

Heart failure (HF) is a common cardiac disorder, affecting millions of people worldwide. Although HF may present at any age, the incidence and prevalence are strongly age dependent. Especially after the fifth decade, HF becomes a very common disorder.^1^ Indeed, above the age of 50, the lifetime risk of developing HF is 1:4. This is true both in men and in women, although generally, HF emerges earlier in men than in women, and some subphenotypes and aetiologies of HF are predominant in men, while others are more prevalent in women.

When considering HF in the elderly, the most immediate question is how to define ‘elderly’. Currently, however, there is no widely accepted consensus on this definition. Instead, arbitrary age cut-offs are often used, e.g. >70–75 years or even above 80 years of age. These dichotomous cut-off values are clearly suboptimal. Furthermore, chronological age represents only one way of expressing the process of ageing, as some subjects may exhibit accelerated biological ageing,^2^ e.g. due to accumulation of lifestyle or environmental factors, such as smoking, excessive alcohol consumption, the presence of other diseases such as renal disease, or the exposure to toxic drugs such as chemotherapy. In fact, HF is characterized by an accelerated biological ageing phenotype of the heart, with key molecular and cellular hallmarks of ageing exacerbated in affected patients.^3,4^ While further research is needed to elucidate the clinical relevance of these hallmarks across HF subtypes, available data strongly implicate key ageing mechanisms, including impaired autophagy and proteostasis, mitochondrial dysfunction, cellular senescence, clonal haematopoiesis of indeterminate potential (CHIP), and inflammation as both contributors to HF pathogenesis and potential therapeutic targets.^5^ As such, a HF subject who has a chronological age of 60 years may have a biological age of a 70-year-old.^6,7^ Thus, in the interest of clarity, reference to age in this review primarily pertains to chronological age, unless stated otherwise.

Contemporary recommendations for optimal HF treatment do not take into consideration the age of affected patients, although efficacy, safety, and tolerability may differ significantly between young and old patients with HF. The majority of the evidence for HF treatment has not been specifically derived from elderly populations. Instead, efficacy and safety estimates are largely extrapolated from younger patients. This reflects the design of most trials, which typically enrol patients with no or few comorbidities and who are fit enough to attend outpatient visits. As a result, most of the clinical evidence is based on a patient population that differs considerably from the average HF patient, leaving significant knowledge gaps in the treatment and general clinical management of elderly patients with HF.

In this review, we will describe the epidemiology of HF in the elderly. Furthermore, we will discuss the most recent insights that have emerged with regard to ageing and changes in the heart, which explain—at least in part—the pathophysiology of HF in the elderly. Finally, we will discuss the evidence for HF drug and device treatment in the elderly and discuss several considerations pertinent to HF management thereof.

## Epidemiology

Heart failure affects over 64 million people worldwide, imposing a major clinical, social, and economic burden. Numerous comprehensive reports provide important insights into its epidemiology.^8,9^ Therefore, our goal herein is to focus on recent material while guiding readers to the original sources for more detailed information.

In Europe, the Heart Failure Association (HFA) of the European Society of Cardiology (ESC) launched the HFA Atlas survey in 2019, covering 43 ESC countries.^10–12^ The initial report revealed significant regional variations in key indicators related to the burden of HF—including prevalence, incidence, mortality, and morbidity—largely influenced by differences in definitions and data sources. The latest findings, published in 2025 and based on data from the 2021–23 European HF Survey, reported a median HF prevalence of 1.9% [interquartile range (IQR) 1.4%–3.4%], with estimates ranging from ≤1.2% in Spain to over 3.0% in Estonia. The median 1-year mortality rate was 14.5% (IQR 8.2%–21.6%), ranging for 4.1% in North Macedonia to over 25% in Bosnia and Herzegovina, Lithuania, and the Netherlands.^10^

In the USA, the *HF Stats 2025: Heart Failure Epidemiology and Outcomes Statistics* revealed that the lifetime risk of HF has increased to 24%. Since 2012, HF-related mortality rates have shown a consistent upward trend.^13^

In ambulatory adults with chronic HF, the estimated 1-year mortality rate is ∼13.5%. Among patients aged 65 years and older who are hospitalized for HF, the estimated 1-year post-discharge mortality rate is 35%. While HF-related mortality increases substantially with advancing age—particularly beyond 74 years—a more pronounced relative annual increase in HF-related mortality has been observed among younger adults aged 35–64 years, compared with those aged 65 to 84 years.

Informal comparisons suggest that lifetime risk, prevalence, and mortality of HF are broadly similar between Europe and the USA. However, standardized and methodologically rigorous data collection is urgently needed to accurately assess disparities and track improvements in care. Reported findings must be interpreted in light of data sources, as reliance on clinical trials or specialty registries may introduce referral bias.

The rise in HF-related deaths is quite concerning, especially given ongoing efforts to reduce its burden. The evidence that survival gains noted until 2010 have markedly eroded,^14^ consistent with data from Europe, is increasingly attributed to the rising prevalence of HF due to population ageing.^6^

### Age-specific incidence, prevalence, and trends of heart failure

**Figure 1:**
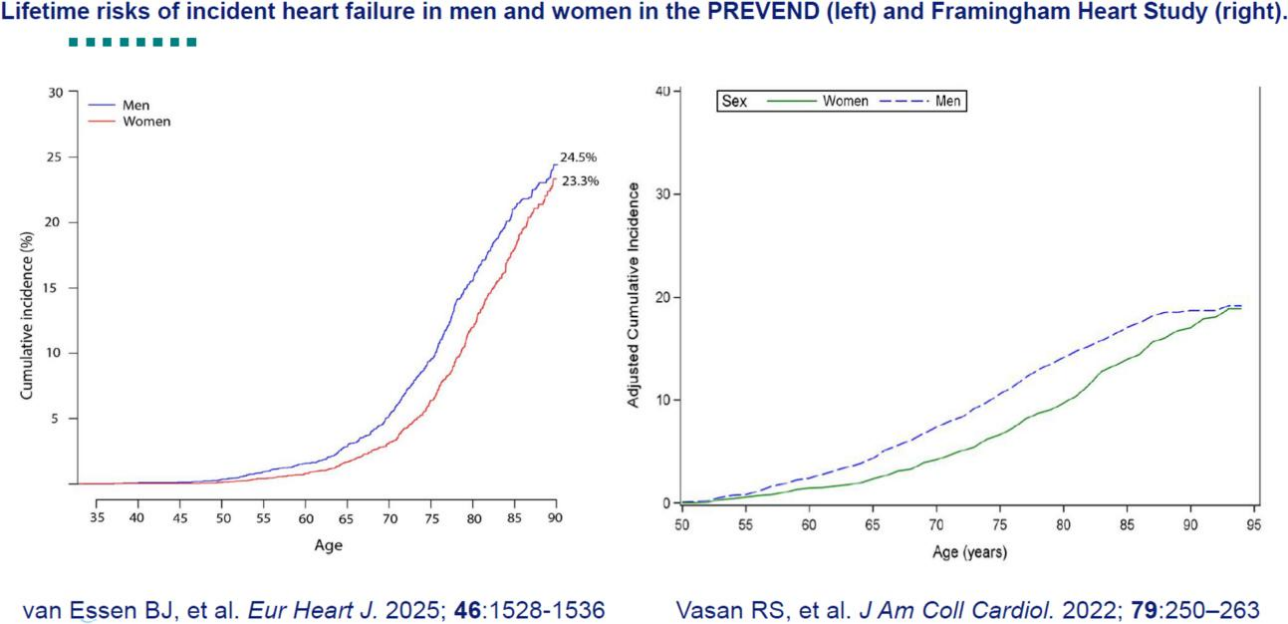
Lifetime risk estimates for incident heart failure. Data from the extended follow-up of the Prevention of REnal and Vascular ENd-stage Disease cohort and the Framingham Heart Study show the lifetime risk for men (blue lines) and women (red lines) for incident heart failure (reprinted with permission)

While there is limited data on trends in age-specific incidence of HF, an important study from Denmark reported on the incidence of HF between 1995 and 2012 among all Danish individuals.^15^ Interestingly, the incidence of HF decreased over the study period among older individuals and conversely increased in younger persons. Importantly, these crude numbers may be influenced by changing patterns of ascertainment, competing risks, and survival, not only by biological shifts in disease occurrence. These trends are observed in the context of an increasing prevalence of comorbidities including, obesity diabetes, hypertension, and atrial fibrillation among younger persons with HF. Clearly, HF is significantly more prevalent as age increases, affecting 4.3% of individuals aged 65–70 in 2012. This rate is expected to rise consistently, potentially reaching 8.5% by 2030.^16^ Due to its high prevalence in the elderly, HF is frequently considered a component of geriatric cardiovascular syndromes, which are often complicated by multiple coexisting health conditions and frailty—factors that greatly increase both the individual and societal burden of the disease.^16^ Most estimates for the lifetime risk to develop either heart failure with reduced ejection fraction (HFrEF) or heartfailure with preserved ejection fraction (HFpEF), and their associated risk factors have been derived from (two) studies from the USA, the Framingham Heart Study (FHS) and the Multi-Ethnic Study on Atherosclerosis (MESA). A recent analysis described the sex-specific lifetime risk and population attributable fraction of potentially modifiable risk factors for incident HFpEF and HFrEF in a large European community-based cohort: 8558 participants from the PREVEND cohort were observed for 25 years for cases of new-onset HFrEF [leftventricular ejection fraction (LVEF) < 50%] and HFpEF (LVEF ≥ 50%) by assessment of hospital records. A total of 804 cases of new-onset HF were identified (534 HFrEF and 270 HFpEF) during 25 years of follow-up. The mean age at onset of HF was 72.1 years in men and 74.2 years in women. The overall lifetime risk of developing HF was 24.5% in men compared with 23.3% in women. The lifetime risk of HFrEF was lower in women compared with men (11.9% vs 18.1%), while the lifetime risk of HFpEF was higher in women compared with men (11.5% vs 6.4%).^1^ Of note, the lifetime incident curves look very similar in the EU and USA (*Figure 1*).

Notably, the LVEF, most often measured in clinical practice by echocardiography, is the cornerstone of the clinical classification of HF despite criticisms of its reproducibility and physiological relevance.^17,18^ Over time, the respective proportion of HFpEF vs HFrEF has shifted with an increase in the proportion of persons classified as having preserved ejection fraction. It remains unclear if this represents a true change in disease epidemiology, or improved recognition and diagnostic sensitivity for HFpEF, or a combination of both. Furthermore, the cut points used to describe a category of HF based on the LVEF have varied over time and across professional societies.^19^ In any way, although the prevalence of HFpEF appears to be rising across all age groups, it remains primarily a condition of the elderly.^20^ Indeed, only 14% of HF diagnoses in individuals under 40 years of age present as HFpEF.^21^ Finally, right ventricular (RV) failure is a frequent phenomenon complicating left-sided HF, also in the setting of HFpEF. The risk factors and pathophysiological mechanisms of RV failure are complex, including pulmonary hypertension, ventricular interdependence, atrial fibrillation, coronary artery disease, and several non-cardiac comorbidities such as obesity, diabetes, and renal dysfunction. Clearly, all of these are more common in elderly, and RV failure is thus more commonly observed in elderly HF patients. Treatment of RV failure is mostly empirical, and no RV-specific therapeutic agents have been developed.^22^

### Risk factors and comorbidities

Hear failure is often the end manifestation of other forms of heart disease and risk factors including hypertension, ischaemic heart disease, and cardiomyopathy, which are particularly frequent in the Western world. The temporal trends in risk factors, particularly hypertension, obesity, diabetes, and atherosclerotic cardiovascular disease, parallel the temporal trends in HF prevalence. In this context, the alarming increase in the prevalence of obesity and diabetes is in keeping with the forecasted increase in the prevalence of HF over time.^9,23^

These trends underscore the urgent need to rethink our approach to the growing HF burden. Shifting towards prevention through risk factor management and addressing health inequities is essential to reduce HF-related morbidity and mortality.

As HF prevalence rises with age, so does the burden of comorbidities, making multimorbidity common among older adults with HF. Notably, its prevalence exceeds what age alone would predict. Indeed, in a community-based case–control study, patients with HF exhibited a higher prevalence of several risk factors compared with HF-free controls.^24^ Cardiometabolic conditions such as diabetes and obesity were more strongly linked to HF in younger individuals.

Recently, the concept of cardiovascular, kidney, and metabolic (CKM) syndrome was proposed, defined by risk factors and established CV disease, with CKM stages ranging from 0 (no risk factors) to 4 (established CVD). Not surprisingly, it has been reported that CKM sharply increases with age. Adults in the 20–44 age group had CKM Stage 3/4 in 2.1% of the cases, but adults 45–64 had CKM Stage 3/4 in 10.7%, while adults ≥65 years had CKM Stage 3/4 in 55.3%.^25^

Comorbidities may precede or develop after HF, and several comorbid conditions usually coexist with HF at the time of diagnosis. Non-cardiovascular comorbidities (anaemia, chronic kidney disease, diabetes mellitus, depression, pulmonary diseases, sleep-disordered breathing, and others) are common and increase morbidity and mortality risk in HF.^26^ It appears these risk factors importantly modify the HF risk in elderly. In a European cohort, in women, 71% of incident HFrEF cases were attributable to eight risk factors (hypertension, hypercholesterolaemia, obesity, smoking, atrial fibrillation, chronic kidney disease, myocardial infarction, and diabetes mellitus) and 60% in men. In women, 64% of incident HFpEF cases were attributable to those risk factors, whereas this was 46% in men. More specifically, in both men and women, hypertension and hypercholesterolaemia were the strongest risk factors for HFrEF, whereas hypertension and obesity were the strongest risk factors for HFpEF.^1^

## Pathophysiological mechanisms of ageing in heart failure

### Impaired autophagy and proteostasis

Macroautophagy (herein referred to as autophagy) is a fundamental homeostatic process essential for the degradation of cytoplasmic proteins and organelles for detoxification, recycling, and energy substrate provision.^27^ As such, autophagy is particularly critical for post-mitotic cells such as cardiomyocytes, which rely on efficient autophagic and proteosomal machinery for survival, as their accumulated cargo cannot be diluted through cell division. Autophagic activity progressively declines with ageing, increasing the risk of organelle dysfunction and proteotoxic stress across various cell types, and pathological conditions.^28^ Specifically in HF, proteostasis analysis of human hearts with dilated or hypertrophic cardiomyopathy have demonstrated significantly reduced proteasomal activity compared with non-failing donor hearts.^29^ Impaired protein turnover and autophagy have been also shown to contribute to cardiomyocyte dysfunction in dilated cardiomyopathy and associated HFrEF.^30,31^ Furthermore, transcriptomic profiling of human myocardial tissue has revealed that autophagy is even more profoundly downregulated in HFpEF, irrespective of associated comorbidities.^32^ By contrast, increased myocardial autophagic activity in patients with dilated cardiomyopathy and HFrEF has been associated with left ventricular reverse remodelling and improved clinical outcomes.^33^ Notably, emerging autophagy-activating interventions, such as spermidine, trehalose, and Tat-Beclin 1, among others, have demonstrated therapeutic efficacy across experimental models of cardiomyopathy and HF.^34–38^ Clinically, restoration of proteostasis using chaperone-mimicking drugs, such as tafamidis and acoramidis, has significantly improved HF outcomes in transthyretin amyloid cardiomyopathy, a prototypical proteotoxicity-driven cardiac disorder that predominantly affects elderly men.^39,40^ However, the efficacy of these protein stabilizers, as well as other autophagy-enhancing strategies, remains to be established in the broader elderly population with HF.

### Mitochondrial dysfunction and oxidative stress

Mitochondrial defects are a hallmark of ageing, affecting nearly every organ system.^41^ However, none is as vulnerable as the heart—the most metabolically active organ, with the highest mitochondrial content of any tissue.^42^ Indeed, cardiomyocytes are densely packed with mitochondria, which occupy nearly one-third of their volume.^43^ Thus, efficient mitochondrial quality control via autophagy-dependent degradation (i.e. mitophagy) is essential to prevent the accumulation of dysfunctional mitochondria,^44^ which otherwise become major sources of oxidative stress and inflammatory signalling.^45^ Consequently, mitochondrial dysfunction is increasingly recognized as both a hallmark and a driver of HF,^42^ not only due to its impact on myocardial bioenergetics but also because of its regulatory roles in cellular stress, survival, and death.^46,47^

Failing hearts exhibit structurally abnormal mitochondria—often fragmented or forming complex aggregates—accompanied by disrupted electron transport chain function and impaired adenosine triPhosphate (ATP) production.^48^ As a result, mitochondrial fatty acid oxidation fails to meet cardiac energy demands, particularly during exercise or other conditions of increased cardiac workload. Consequently, the failing heart shifts towards glycolytic metabolism, especially in late-stage disease.^49^ Although mitochondrial dysfunction is evident across the spectrum of HF phenotypes, distinct metabolic differences exist between HFpEF and HFrEF.^50^ In HFrEF, fatty acid oxidation is more severely impaired, as indicated by long-chain acylcarnitine accumulation,^51^ whereas HFpEF is associated with a more pronounced mitochondrial dysfunction in skeletal muscle, reflected by rapid depletion of high-energy phosphates during exercise.^52^ These structural and functional mitochondrial impairments contribute to a maladaptive cycle that drives disease progression. Therefore, various aspects of mitochondrial health have been explored for therapeutic targeting.

For instance, the mitochondria-targeted antioxidant SS-31 (elamipretide) improved redox balance and cardiac function in aged mice as well as preclinical models of HF.^53,54^ Elamipretide also restored mitochondrial function in hypertrophic cardiomyopathy patient-derived tissues.^55^ However, a Phase II trial in patients with HFrEF failed to demonstrate clinical benefit,^56^ and the compound remains under investigation in HFpEF (NCT02814097). Similarly, the antioxidant MitoQ showed cardioprotective effects in a preclinical HF model,^57^ but its efficacy in patients remains to be proven. Beyond redox modulation, mitochondrial energy metabolism has also been directly targeted. For instance, coenzyme Q10—an electron transport chain component that enhances ATP production by engaging in redox reactions—has demonstrated efficacy as an adjuvant therapy in patients with HFrEF,^58^ though not in HFpEF.^59^ Interestingly, the benefits of coenzyme Q10 in HFrEF were reported to be more pronounced in older patients (>67 years), in line with the rationale of personalizing the treatment of elderly patients with HF. However, the aggregate data comprise a limited number of patients, and no definitive recommendations with regard to Q10 supplementation can be provided.^60^ Nicotinamide adenine dinucleotide (NAD), a key redox cofactor essential for mitochondrial function, is yet another major therapeutic target. Indeed, cardiac NAD levels decline with ageing and other major cardiovascular risk factors,^61^ whereas NAD replenishment—using nicotinamide or nicotinamide riboside—demonstrated efficacy in preclinical models of both HFpEF and HFrEF.^62,63^ Besides enhancing cardiac and skeletal muscle bioenergetics, the cardioprotective effects of NAD were also shown to depend on autophagy activation.^64,65^ In patients with HFpEF, increased nicotinamide degradation correlated with worse outcomes,^64^ while nicotinamide riboside supplementation improved mitochondrial respiration and reduced inflammation in HFrEF.^66^ Thus, large clinical studies are warranted to test the efficacy of NAD precursors in patients with HF.

### Inflammaging, cell senescence, and clonal haematopoiesis of indeterminate potential

Ageing is associated with chronic low-grade inflammation, known as inflammaging.^67^ This is reflected in the ageing heart by a significant increase in immune cell infiltration and activation.^68^ Similarly, failing hearts exhibit increased infiltration of macrophages, dendritic cells, and CD3⁺ T cells, which contribute to adverse remodelling and poor prognosis.^69,70^ Systemically, circulating inflammatory markers—particularly interleukin-6 and tumour necrosis factor-α—are increased and independently associated with incident HF in older adults.^71^ While inflammation is more strongly linked to HFpEF due to its predominantly aged and comorbid patient population,^72^ substantial evidence implicates inflammatory processes in HF pathogenesis across the LVEF spectrum.^71,73^ Despite this strong pathophysiological basis, most clinical trials targeting inflammation in HF have been largely unsuccessful, with only a few exceptions (reviewed here^74^). This underscores the need for more personalized and mechanism-based immune-modulatory therapeutic approaches.

In this context, multiple mechanisms have been proposed to drive inflammation in HF^73^ and ageing.^67^ Among them, cell senescence and CHIP stand out as shared contributors, potentially playing a key role in elderly patients with HF. Cell senescence refers to a state of permanent cell cycle arrest, accompanied by the secretion of a range of pro-inflammatory cytokines and chemokines, collectively termed the senescence-associated secretory phenotype (SASP). Notably, several SASP factors correlate with disease severity and adverse outcomes in both HFrEF and HFpEF.^75,76^ In preclinical models, accelerated endothelial senescence exacerbated HFpEF development,^77^ while treatment with senolytic agents (compounds that selectively eliminate senescent cells) exerted cardioprotective and anti-inflammatory effects in HFrEF.^78,79^ These epidemiological and experimental findings support the need for clinical trials to evaluate the therapeutic potential of senescence-targeting strategies in HF.^80^

Clonal haematopoiesis of indeterminate potential, characterized by the presence of somatic mutations in the blood of otherwise healthy older individuals, is another age-related pro-inflammatory mechanism strongly linked to HF. Clonal haematopoiesis of indeterminate potential mutations affect up to 40% of individuals over 70 years old and confer a cardiovascular risk comparable to major risk factors such as hypertension, Type 2 diabetes, smoking, and elevated cholesterol.^81^ Specific CHIP mutations, particularly *ASXL1*, *TET2*, and *JAK2*, are associated with a 25% increased risk of new-onset HF.^82^ Moreover, *TET2* mutations have been linked to a higher incidence of HFpEF, but not HFrEF,^83^ though other studies have demonstrated that CHIP mutations independently predict poor prognosis and adverse outcomes in both HFpEF and HFrEF.^84–86^ Intriguingly, early CHIP development in individuals under 65 years is significantly associated with incident HF,^87^ reinforcing the concept that accelerated biological ageing contributes to HF. Supporting this, young mice with CHIP-associated *TET2* mutations exhibit exaggerated cardiac dysfunction and adverse remodelling in preclinical models of HFpEF and HFrEF.^86,88^ Mechanistically, these effects are mediated by NLRP3/interleukin-1β–driven pro-inflammatory activation. However, further research is needed to develop viable strategies for targeting CHIP in patients.

**Figure 2:**
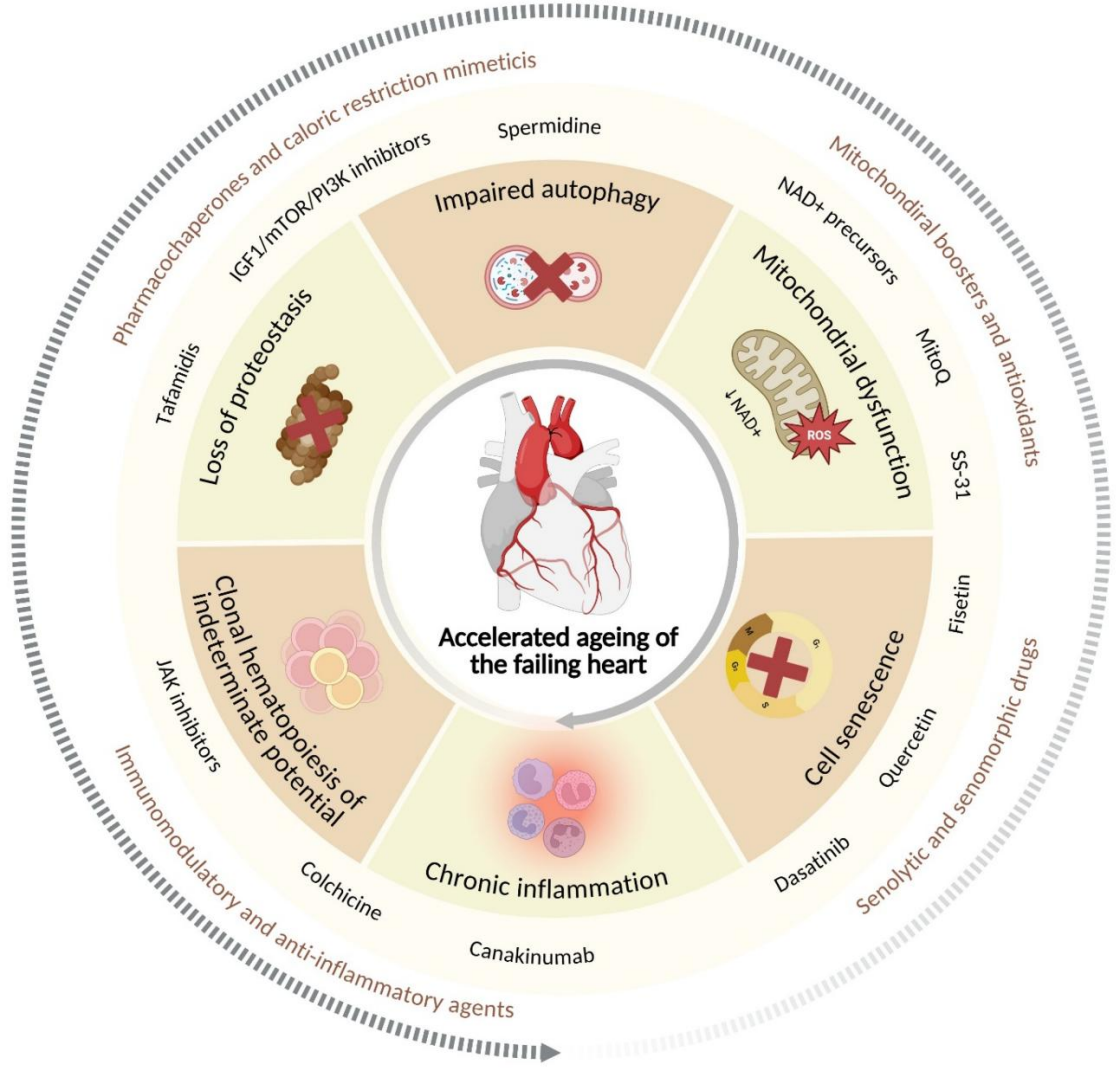
Molecular and cellular mechanisms of ageing in heart failure. Key hallmarks of ageing within the cardiovascular system are depicted, including impaired autophagy and proteostasis, mitochondrial dysfunction, and oxidative stress, as well as cell senescence, chronic inflammation, and clonal haematopoiesis of indeterminate potential. These mechanisms are exacerbated in the failing myocardium, denoting an acceleration of biological ageing in the context of heart failure. This suggests that therapeutic strategies targeting cardiovascular ageing may hold promise for improving outcomes in elderly patients with heart failure. A non-exhaustive list of examples is shown, including clinically approved and emerging experimental drugs with potential relevance to this population. IGF1, insulin-like growth factor 1; mTOR, mechanistic/mammalian target of rapamycin; NAD, nicotinamide adenine dinucleotide; PI3k, phosphoinositide 3-kinase; ROS, reactive oxygen species

The molecular and cellular mechanisms of ageing in HF are summarized in *Figure 2*, and a regularly updated list of ongoing or completed trials testing the efficacy of senolytic agents across the spectrum of age-related diseases is accessible here: https://www.gerosciencenetwork.org/clinical-trials-data.

## Safety and efficacy of guideline-recommended medical therapy in elderly patients with heart failure

Most randomized clinical trials (RCTs) in HF have been conducted in relatively young, male patients with HFrEF. Notably, female patients and patients with heartfailure with mildly reduced ejection fraction (HFmrEF) and HFpEF are generally older than the typical male patient with HFrEF. Therefore, gaps in knowledge exist in elderly patients, in female patients, and in patients with HFmrEF and HFpEF.

However, given the number and large size of several RCTs in the HF space, the proportion of patients who are >75 or even >80 years of age is still considerable, and several hallmark trials have reported in separate papers on the efficacy and safety of HF drugs specifically in the elderly. In *Table 1*, we present the efficacy of the four HF pillars from large individual-data meta-analyses: renin–angiotensin–aldosterone system (RAAS) inhibitors, beta-blockers, mineralocorticoid receptor antagonists (MRAs), and sodium–glucose co-transporter 2 (SGLT2) inhibitors. Overall, efficacy of these drugs is equal between younger and elderly patients, and in all meta-analyses, the interaction terms between effect size and age were non-significant, demonstrating that there is no interaction of their efficacy with increasing age. Data for the direct stimulator of soluble guanylate cyclase (sGC) vericiguat suggest that the efficacy also for this drug class is preserved across age categories (including elderly), although the numbers are much smaller than for the four main GRMT pillars.^94^

**Table 1:** Efficacy of the four heart failure pillars for younger and older subjects

Class of drugs	Name of the drug	Lead trial(s)	Efficacy young vs elderly patients	Ref
ACE inhibition (ACEi)	Enalapril	SOLVD Consensus	**AC mortality/HHF** < 60 years: .71 (.59–.86) ≥ 60 years: .79 (.66–.95)	^89^
Beta-blockers	Carvedilol Metoprolol Bisoprolol	US Carvedilol COPERNICUS MERIT-HF CIBIS	**CV death/HHF** Q1 (50 years): .66 (.56–.77) Q2 (60 years): .78 (.68–.89) Q3 (68 years): .72 (.62–.82) Q4 (75 years): .89 (.78–1.02)	^90^
Angiotensin receptor–neprilysin inhibitor	Sacubitril valsartan	PARADIGM HF	**CV death/HHF** < 55 years: .78 (.64–.96) ≥ 75 years: .86 (.72–1.04)	^91^
Mineralocorticoid receptor antagonist (MRA)	Spironolactone Eplerenone Finerenone	RALES, TOPCAT EMPHASIS-HF FINEARTS	**CV death/HHF** RALES/EMPHASIS Age < 75 years: .66 (.58–.74) Age ≥ 75 years: .66 (.53–.82) TOPCAT/FINEARTS Age < 75 years: .84 (.75–.95) Age ≥ 75 years: .89 (.78–1.02)	^92^
Sodium–glucose co-transporter 2 inhibitor (SGLT2i)	Dapagliflozin Empagliflozin	DAPA-HF DELIVER EMPEROR-Reduced EMPEROR-Preserved	**CV death/HHF** Age < 65 years: .79 (.70–.87) Age ≥ 65 years: .77 (.71–.83)	^93^

Key individual-data meta-analyses of large RCTs were studied and results are displayed (for sacubitril/valsartan, only data from PARADIGM-HF were considered). Results are shown as hazard ratio (HR) with 95% confidence intervals compared with placebo (or compared with enalapril for sacubitril/valsartan).

ACE, Angiotensin-converting enzyme; CV, cardiovascular; AC, all cause; HHF, hospitalization for heart failure.

Furthermore, a number of (more recent) trials have enrolled a relatively elderly population, especially those with HFmrEF and HFpEF. For instance, the Irbesartan in Heart Failure with Preserved Systolic Function (I-PRESERVE) trial (mean age 73 years),^95^ the Study of the Effects of Nebivolol Intervention on Outcomes and Rehospitalisation in Seniors with Heart Failure trial (SENIORS, mean age 76 years),^96^ the Perindopril in Elderly People with Chronic Heart Failure study (PEP-CHF, mean age 76 years),^97^ the Prospective Comparison of ARNI with ARB Global Outcomes in HF With Preserved Ejection Fraction (PARAGON-HF, mean age 73 years),^98^ and the Dapagliflozin Evaluation to Improve the Lives of Patients With Preserved Ejection Fraction Heart Failure trial (DELIVER, mean age 72 years, with and age range of 40–99 years old)^99^ were all trials that enrolled a substantial proportion of elderly patients. As such, contemporary HF trials are placing greater emphasis on participant age to ensure more accurate representation of the general HF population.

In contrast to efficacy, age-related safety parameters are less frequently published; see *Table 2*. Furthermore, the elderly patients included in these studies may only partially resemble the elderly patients in the population, as several aspects of the elderly such as frailty, immobility, and comorbidities may preclude inclusion in randomized trials.

**Table 2:** Safety aspects of the four heart failure pillars for younger and older subjects

Class of drugs	Name of the drug	Lead trial(s)	Efficacy young vs elderly patients	Ref
ACE inhibition (ACEi)	Enalapril	SOLVD Consensus	**Age-related safety not reported**	^89^
Beta-blockers (BBs)	Carvedilol Metoprolol Bisoprolol	US Carvedilol COPERNICUS MERIT-HF CIBIS	**Discontinuation of study treatment** Q1 (50 years): 14.5% (PLC), 12.1% (BB) Q2 (60 years): 14.6% (PLC), 12.7% (BB) Q3 (68 years): 16.1% (PLC), 14.6% (BB) Q4 (75 years): 15.6% (PLC), 14.4% (BB)	^90^
Angiotensin receptor–neprilysin inhibitor (ARNi); comparator ACE inhibitor (enalapril)	Sacubitril valsartan	PARADIGM-HF	**Any side effect leading to study drug discontinuation** < 55 years: S/V: 14 (1.7%) and Ena: 16(2%) ≥ 75 years: S/V: 22 (2.8%) and Ena: 35(4.5%)	^91^
Mineralocorticoid receptor antagonist (MRA)	Spironolactone Eplerenone Finerenone	RALES, TOPCAT EMPHASIS-HF FINEARTS	**Age-related safety not reported**	^92^
Sodium–glucose co-transporter 2 inhibitor (SGLT2i)	Dapagliflozin Empagliflozin	DAPA-HF DELIVER EMPEROR-Reduced EMPEROR-Preserved	**Age-related safety not reported**	^93^

Key individual-data meta-analyses of large RCTs were studied and results are displayed (for sacubitril/valsartan, only data from PARADIGM-HF were considered). Results are shown as hazard ratio (HR) with 95% confidence intervals compared with placebo (or compared with enalapril for sacubitril/valsartan).

PLC, placebo; Ena, enalapril.

There are, however, several suggestive signals that the relative efficacy may decline with increasing age. This has been ascribed to accumulation of comorbidities in the (very) elderly. Clearly, agents that attenuate HF progression cannot be effective in subjects in whom a larger proportion of the risk is no longer explained by HF. Furthermore, side effects of standard HF medication, e.g. hyperkalaemia during treatment with MRA, may arise more frequently in elderly patients who display more renal disease than younger ones.^100^

A major barrier to GRMT in the elderly is (the fear of) low blood pressure. However, as hypertension and even mildly elevated blood pressure (>120 mmHg) are important risk factors for incident HF and progression of HF, often the problem is the anxiety of the treating physicians, but not the real blood pressure *per se*, which frequently is in the normal range and asymptomatic. A dedicated consensus document from the HFA of the ESC provides a comprehensive overview of low blood pressure in HFrEF, including its definition, risk factors, and effects of HF therapies. Management pathways are proposed to optimize HFrEF treatment in the context of low blood pressure, aiming to improve patient outcomes.^101^

Elderly patients often suffer from kidney dysfunction, which is another perceived reason for not optimizing HF therapies.^102^ However, most GRMTs for HF also have—after an initial drop of estimated glomerular filtration rate (eGFR)—a prolonged positive effect on preserving kidney function and preventing end-stage renal disease and dialysis. In fact, the initial eGFR dip is an indicator for these patients who appear to display the greatest absolute benefit from GRMT.^103^ Collectively, although side effects are more common in elderly,^100^ GRMT should always be attempted and established whenever possible irrespective of age; and elderly HFrEF patients tolerating (standard) dosages of GRMT appear to have a better prognosis than patients without or at only low dosages of GRMT.

## Device therapy and heart transplantation in elderly

In addition to GRMT therapy, which should be optimized also in elderly HF patients whenever possible, this population may also profit from certain devices, but they are often excluded from such interventions.^104^

By contrast, valvular interventions, such as transcatheter aortic valve implantation (TAVI) and transcatheter edge-to-edge repair (TEER) for mitral or tricuspid regurgitation (M-TEER, T-TEER), are especially dedicated to aged patients with higher operative risk^105^ and improve outcomes such as mortality/hospitalization (TAVI and M-TEER) and/or quality of life (TAVI, M-TEER, and T-TEER). Thus, advanced age should not be a reason for exclusion from advanced interventional valvular therapies.

Heart replacement therapies such as heart transplantation (HTx) and left ventricular assist devices (VADs) are mainly reserved for younger patients, although in some cases, the age limit has exceeded 70 years.^106^ Although the risk of stroke has decreased significantly with the novel HeartMate 3 leftventricular assist device (LVAD),^107^ the risk of bleeding remains significant. Given the proof that aspirin omission from combined antithrombotic therapy with vitamin K antagonists in HeartMate 3 LVAD patients reduces bleeding but does not increase ischaemic events (ARIES),^108^ LVAD implantation might become a more accessible option for older patients in the future.

Elderly patients with HFrEF may also have symptomatic improvement with baroreflex activation therapy (BAT) and cardiac contractility modulation (CCM); however, the lack of guideline recommendations for these therapies and rather high costs may limit their application in the elderly population.

The most important questions regarding elderly HF patients and device therapies clearly relate to cardiac resynchronization systems (CRTs) and/or implantable cardioverter-defibrillators (ICDs). In HFrEF patients who are likely to derive a large symptomatic benefit from CRT (especially left bundle branch block and QRS duration> 150 ms), even advanced age should not distract from its implantation. A much more individual approach is necessary in elderly patients (especially above the age of 85, or >80 with significant comorbidities) with an EF < 35% regarding the decision to implant an ICD for prevention of sudden cardiac death (SCD).^109^ Given the results of the HF-OPT study, which demonstrated that many patients with newly diagnosed HFrEF (both ischaemic and non-ischaemic ) display significant improvement in EF after 6 months of GRMT intensification,^110^ optimizing GRMT is key before considering ICD implantation.

In elderly patients with an ICD in place, when it comes to a replacement of the battery or when the patient deteriorates with repeated decompensations, or additional life-limiting comorbidities are diagnosed, deactivation of the defibrillator function while maintaining the pacemaker function of the ICD should always be discussed with the patient.^111^

## Special considerations in elderly (*Table 3*)

Some aetiologies of HF are particularly common in the elderly. First, HFpEF is more common than HFrEF in the (very) elderly. A meta-analysis revealed age-dependent increase in left ventricular (LV) diastolic dysfunction, but not systolic dysfunction.^112^ Furthermore, as discussed, the lifetime risk estimates of HF show a clear and sharp increase in incident HFpEF in subjects >70 years of age. Second, amyloid is a condition of disturbed protein folding, and cardiac involvement is often present, especially male elderly subjects, characterized by LV hypertrophy, conduction disturbances, and diastolic dysfunction. Recent registries indicated cardiac amyloid is quite common in patients presenting with HFpEF.^113,114^ The field of cardiac amyloid has seen many new therapies, as discussed extensively recently.^115^

Some HF drugs are used more frequently in elderly patients. In patients with HFrEF and/or atrial fibrillation, digitalis glycosides such as digoxin and digitoxin are still of value, especially in the context of low blood pressure.^116^ In the currently recommended dosages (lower than previously), digitalis glycosides appear to act less as positive inotropes, but preferentially as sympatholytics, and they may be associated with improved symptoms and quality of life. Special care is needed to avoid overdose in the elderly and in patients with kidney dysfunction.^117^ In this context, two studies evaluate digoxin and digitoxin in patients with HF.^118,119^ In contrast to the initial expectations, DIGIT-HF did not include more elderly patients than contemporary HFrEF studies.^120^ Nevertheless, DIGIT-HF results show a significant reduction of the primary endpoint of total mortality/first hospitalization in patients well treated with HFrEF GRMT, without heterogeneity according to age.^121^ As digitoxin elimination is much less dependent on the kidney than digoxin, digitoxin may constitute a very helpful drug in elderly patients with HFrEF.

The ESC and ACC/AHA HF guidelines provide no specific recommendations for the treatment of elderly patients. Most HF treatments are indicated to increase survival, yet quality of life is not necessarily improved. However, for elderly and frail patients, quality of life often is more precious than longevity.^122^ Neurological and psychiatric comorbidities, especially depression, are frequent and need to be considered.^123^ In patients with progressive cognitive and physical decline, especially when living in a nursery home and dependent on help for daily care, palliative care with a shift from prognostic treatment goals to comfort and quality of life is mandatory. Clearly, medications preventing decompensations such as (lower dosages) of diuretics, SGLT2i, and other GRMT can be maintained if well tolerated. Despite the progress in HF management, mortality remains substantial. Therefore, all cardiology societies have adopted palliative care as an integral part of the care pathway. It is therefore recommended to discuss palliative care with elderly patients early, before their condition becomes severe enough to warrant such care.^124–127^ End-of-life discussions should also include deactivation of ICD functions if not done before.^128^

## Practical clinical impact

**Table 3:** Specific considerations in elderly HF patients

Clinical observation or consideration	HF population overall	HF in the elderly
Risk for HF	High lifetime risk	Extremely high risk
HF phenotype Ataeiologies	HFpEF = HFrEF Regular work-up	HFpEF > HFrEF Same, but also consider amyloid
Efficacy of GRMT	Very effective	Very effective, with lower relative risk reductions, but preserved absolute risk reduction
Special drugs	GRMT	GRMT, consider cardiac glycosides
Safety of GRMT	Relatively safe	More safety issues, sometimes necessitating dose reductions
Device therapy	According to guidelines	Personalized decision-making, especially for ICD, accounting for competing risks and comorbidities Discuss non-replacing when end of life and shutting off

## Summary

Heart failure is prevalent in elderly patients, and its incidence and prevalence are expected to rise further. Specific changes occur in the myocardium in response to ageing-associated phenomena. Pharmacotherapy and device therapy have no age-specific restrictions, and therefore clinical decision-making in principle is equal irrespective of age; however, given the abundancy of comorbidities and drug intolerances, tailored decisions must be made. Finally, advanced disease planning and end-of-life decisions are an integral part of HF in the elderly.

## Declarations

### Data Availability

No data were generated or analysed for or in support of this paper.
